# Lipid-Based Drug Delivery Systems in Regenerative Medicine

**DOI:** 10.3390/ma14185371

**Published:** 2021-09-17

**Authors:** Nina Filipczak, Satya Siva Kishan Yalamarty, Xiang Li, Muhammad Muzamil Khan, Farzana Parveen, Vladimir Torchilin

**Affiliations:** 1 Center for Pharmaceutical Biotechnology and Nanomedicine, Northeastern University, Boston, MA 02115, USA; nin.filipczak@northeastern.edu (N.F.); yalamarty.s@northeastern.edu (S.S.K.Y.); xiang.li@jxutcm.edu.cn (X.L.); farzanaparveenphd@gmail.com (F.P.); 2 State Key Laboratory of Innovative Drug and Efficient Energy-Saving Pharmaceutical Equipment, Jiangxi University of Chinese Medicine, Nanchang 330006, China; 3 Department of Pharmaceutics, Faculty of Pharmacy, The Islamia University of Bahawalpur, Punjab 63100, Pakistan; muzamilpharmacist@gmail.com; 4 Department of Oncology, Radiotherapy and Plastic Surgery, I.M. Sechenov First Moscow State Medical University, 119991 Moscow, Russia

**Keywords:** lipid nanoparticles, regenerative medicine, colloids, micelles, drug delivery systems, gene delivery, protein delivery

## Abstract

The most important goal of regenerative medicine is to repair, restore, and regenerate tissues and organs that have been damaged as a result of an injury, congenital defect or disease, as well as reversing the aging process of the body by utilizing its natural healing potential. Regenerative medicine utilizes products of cell therapy, as well as biomedical or tissue engineering, and is a huge field for development. In regenerative medicine, stem cells and growth factor are mainly used; thus, innovative drug delivery technologies are being studied for improved delivery. Drug delivery systems offer the protection of therapeutic proteins and peptides against proteolytic degradation where controlled delivery is achievable. Similarly, the delivery systems in combination with stem cells offer improvement of cell survival, differentiation, and engraftment. The present review summarizes the significance of biomaterials in tissue engineering and the importance of colloidal drug delivery systems in providing cells with a local environment that enables them to proliferate and differentiate efficiently, resulting in successful tissue regeneration.

## 1. Introduction

Regenerative medicine is an interdisciplinary branch of science covering medical biology, biotechnology and biophysics. First coined in 1999 by William Hasetine, he referred to tissue engineering as creating organs or tissues *in vitro* [1]. Its main goal is to develop and apply methods to improve the structure and function of tissues and organs that have deteriorated because of disease development, trauma, or aging. This trend includes both tissue engineering projects aimed at obtaining organs for transplantation in laboratory conditions as well as research on stem cells, which under physiological conditions play key roles in tissue regeneration as well as genetic engineering, drug delivery and materials science [2,3]. Regenerative medicine offers hope for healing from diseases previously considered incurable. So far, its effectiveness has been confirmed in only a few clinical indications, for example, in haemopoietic disorders and skin diseases [3]. Despite some difficulties related to the commercialization of cell-based therapies, bone-marrow-derived stem cells have been used successfully in the clinic for bone, cartilage, spinal cord, cardiac, and bladder regeneration [2], which were the only clinically approved stem cell therapies until 2015 [4]. Over the past decade, there has been rapid growth in the field of experimental regenerative medicine therapies, mostly triggered by the advances in stem cell biology, especially the discovery that mature cells can be reprogrammed to form pluripotent stem cells [5]. However, so far, only a few indications have been confirmed, usually for rare or very rare diseases [6]. Stem cell transplantation is intended to replace lost cells, such as neurons in the brain or beta cells in the pancreas, which requires the transplanted cells to reach their destination and integrate functionally into tissue after differentiation. This approach may provide trophic support, short- or long-term support for the growth, differentiation, or survival of cells with secreted substances. It may also affect immunomodulation or increase plasticity, functions that are indirect and, therefore, often difficult to measure in patients. Reports on immunomodulatory or other difficult-to-quantify measures of stem cell functions have generated controversy [7].

**Figure 1:**
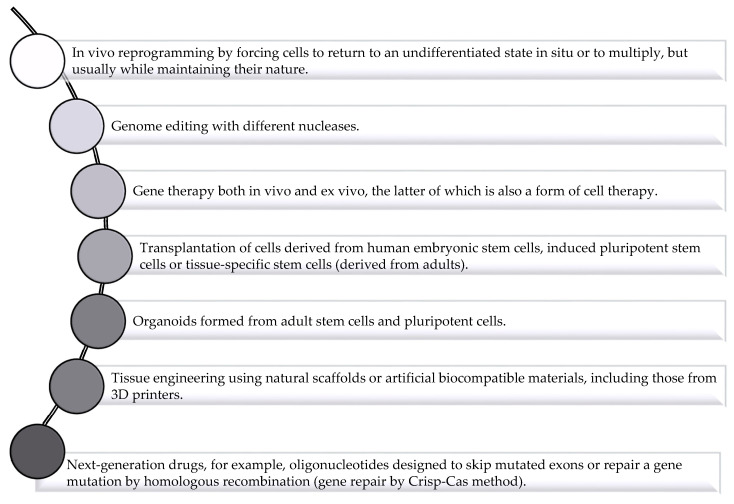
Regenerative medicine: different types of therapy (based on [3]).

Regenerative medicine offers much hope for overcoming difficult-to-treat diseases, especially those affecting the aged population, and for reducing healthcare costs [8]. There are also applications for treating more common diseases such as age-related vision loss or corneal burns, although treatment for these conditions has so far been used only in a small number of patients, mostly in clinical trials. Until now, only two companies have been widely known in the field of regenerative medicine: Organogenesis (specialized in wound healing and regeneration therapies) and Medtronic (specialized in cardiac and vascular disease therapies, neurological and musculoskeletal conditions and diabetes) [9]. Although cell-based therapy is the best known in the field of regenerative medicine, the outcome of tissue regeneration is associated with the drugs, proteins and even genes that can affect the fate of cells. Therefore, the therapies based on the delivery of these agents, as shown in Figure 1, are considered as regenerative medicine [3,10]. The difficulty with therapies based on active agents consists in the delicate nature of these compounds, their short half-lives, and a requirement for intracellular delivery [11]. This is where the development of delivery systems become a promising strategy to overcome the limitations. This review will specifically focus on lipid-based delivery systems used in regenerative medicine.

## 2. Application of Drug Delivery System to Solve Challenges Facing Regenerative Medicine

The human body has three fundamental elements that are essential for regeneration: the extracellular matrix (ECM) as a natural scaffold for cell proliferation and differentiation, plus cells and molecules responsible for signaling. Regenerative medicine utilizes these features singly or in combination. Regenerative therapies can be thought of as having two strands, in vitro or in vivo, depending on the site where tissue regeneration or organ replacement is performed. In vitro regenerative medicine is based on the reconstruction of tissues by means of cell culture methods and the replacement of organs with functional cells, called bioplastic hybrid organs. Currently, this approach is difficult to implement because the biological environment for tissue reconstruction is highly complex and current tissue culture techniques have not allowed for its complete reconstruction.

Unlike in vitro regenerative therapy, in vivo therapy is advantageous for inducing tissue regeneration, as most of the biological components necessary for tissue regeneration, such as growth factors and cytokines, are naturally present in the body. In in vivo regeneration therapy, tissue regeneration is typically achieved using implantation of a biodegradable scaffold, with or without cells [12]. In this case, active and immature cells infiltrate the biodegradable scaffold matrix from surrounding healthy tissue to form new tissue. This therapy is usually effective when patients are young and healthy, and the damaged tissue has a high potential for regeneration. This approach can provide site-specific drug delivery but often requires invasive procedures for placement [13].

On the other hand, if the patients are older and/or suffer from other diseases, such as diabetes and hyperlipemia, which lowers the regenerative potential of the tissue, it is necessary to deliver the growth factor to the regeneration site. In this case, direct injection of a growth factor or any other signaling molecules into a damaged site is usually not effective since they can easily diffuse from the injected site and can be quickly degraded or deactivated [14].

**Figure 2:**
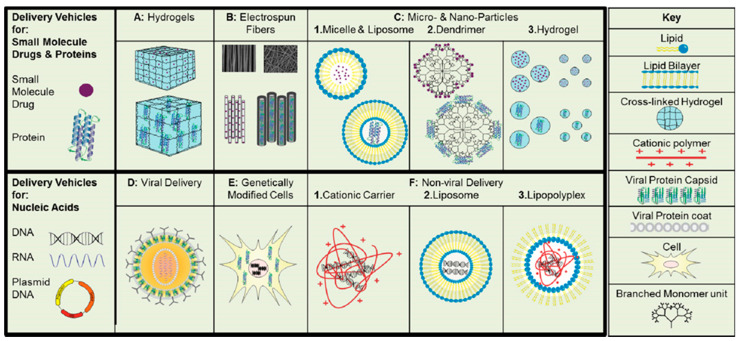
Types of the delivery vehicles used in regeneration therapies for soluble factors. Macroscopic carriers: hydrogels (**A**) and electrospun fibers (**B**). Microscopic carriers: liposomes and micelles (**C.1**), dendrimers (**C.2**), or particulate hydrogel systems (**C.3**). Viral carriers (**D**) and genetically modified cells (**E**). Non-viral delivery vehicles: cationic polymers (**F.1**), liposomes (**F.2**) and lipopolyplexes (**F.3**) [14].

Recently, nanotechnology has been utilized to solve some issues related to tissue regeneration. The most important approach to finding a fitting solution for tissue repair is to understand the physiology of the deteriorated tissue and the pharmacology of the agents needed [15]. Thus, there is no singular solution for tissue regeneration. The choice of the soluble compound that needs to be delivered, as well as the carrier, mostly depends on the injured tissue’s physiology. For example, for growth factors, the delivery system must provide a sustained, controlled release in the area of tissue damage. For that, the best candidates are liposomes, dendrimers or hydrogels, as shown in Figure 2. The nanoparticles mentioned above have the ability to entrap drug molecules suitable for a specific disease indication. Moreover, they also must be relatively easy to be surface modified, according to the desired properties or targeting site [14,15,16].

Similar to the growth factor delivery, the active molecules used in cardiovascular tissue regeneration utilize the same features of the drug delivery systems and can be injected intravenously, administered orally or introduced by pulmonary inhalation. Their nanoscale allows them to reach almost every tissue and allows for limited clearance from the blood by macrophages [13,16]. The cardiovascular system has several therapeutic targets ranging from myocardial infarction (heart attack) and atherosclerosis to ischemic/reperfusion injury, which require different treatment approaches. A form of a therapy to enhance myocardial salvage after successful reperfusion is based on the usage of lipids and detergent emulsions containing perfluorochemicals (Fluosol), which preserves the endothelial structure and endothelium-dependent relaxation of large and small vessels [17].

In some cases, the drug delivery system may help to distribute the active compound to various target organs, such as the brain. The brain microvasculature, containing the blood–brain barrier (BBB), is selectively permeable due to the presence of tight junctions. Because of the size limits of the tight junctions, only transporter-mediated or transcellular uptake into the brain can occur. Interestingly, a recent study found that the adenosine receptor A2A may open the BBB. This finding may serve as a Trojan horse or stealth technology for the delivery of compounds to the brain [13].

Gene therapy in which plasmid DNA or adenovirus is injected directly to produce a therapeutic effect is one of the newest techniques used to aid tissue regeneration. Unfortunately, nucleic acids such as RNA, DNA and plasmid DNA are not very stable after injection, and their negative charge is an obstacle to crossing the negatively charged cellular membrane. Thus, there is a special need for a delivery system for these types of cargo. The unique design requirement for the nucleic acids carriers is the ability to carry that negatively charged cargo and facilitate the cellular uptake [14]. There are two possible ways that delivery systems can help in tissue regeneration based on the delivery of nucleic acid. One way is to deliver cargo to cells by carrier injection to the site of action. Once cells uptake it, they are able to produce the required growth factor for some time and thus facilitate the tissue regeneration [18]. The other option is to genetically modify the cells to produce specific proteins before implantation to the damaged site [19].

In summary, delivery systems play a supportive role in regenerative medicine, which is mainly based on tissue engineering. Delivery systems in tissue regeneration are tools used to help to deliver sensitive compounds to the target site and protect them against degradation. The nanotechnology development offers a variety of solutions for tissue regeneration based on the properties of the cargo and the type of the tissue.

## 3. Materials Used in Regenerative Medicine

The functional replacement of the damaged tissue or organ utilizes naturally occurring or synthetic scaffolding materials that facilitate the attachment, structural support, maintenance, proliferation and differentiation of selected cell populations. The characteristics of scaffolding materials such as shape, composition, mechanical properties and abilities that suppress the host response play a vital role. The highly porous scaffolding biomaterials act as templates and react with body cells for guided tissue growth. The various key considerations for the suitability of a scaffold for human tissue regenerative purposes are important for clinical translation [20]. The biomaterials utilized for the successful fabrication of a scaffold should be carefully selected based on the following listed characteristics.

- It should be biocompatible with no or minimal immune response in order to prevent body rejection caused by inflammatory responses due to cytotoxicity, thrombogenicity, mutagenicity and genotoxicity [21,22,23].
- It must be biodegradable, allowing target cells to produce their own extracellular matrix [24,25].
- It should have sufficient mechanical integrity until the completion of the remodeling process [26,27].
- It must have a critical mean pore size to allow for the transport of nutrients and waste products around the injured tissue [28,29].
- The manufacturing process should be cost-effective and scalable to good manufacturing standards (GMP) [30].
- The preferable off-shelve availability without involving extra surgical procedures is also an important criterion to the choice of biomaterials [31,32].

The biomaterials are classified mainly into three groups, namely, natural polymers, synthetic polymers and ceramics (hydroxyapatite (HA) and tricalcium phosphate (TCP)). The natural biomaterials are protein-based (collagen, gelatin, fibrin, silk), polysaccharides (chitosan, starch, alginate, hyaluronic acid) and polyesters (polyhydroxyalkanoates (PHA)). These polymers present excellent biocompatibility, biodegradability, cell adhesion and growth ability. However, poor mechanical strength and uncontrolled degradation rates are major drawbacks for naturally occurring polymers utilized to construct scaffolds, especially for most orthopedic regeneration procedures. The composite scaffolds with an added ceramic phase or synthetic polymers with natural polymers have been tested for enhanced biological and mechanical properties by various research groups [33,34,35,36,37,38]. Table 1 shows a summary of different biomaterials, fabrication methods properties and their applications.

The synthetic polymeric biomaterials include saturated aliphatic polyesters (poly(glycolic acid) (PGA), poly(lactic acid) (PLA), poly(lactic-co-glycolic acid) (PLGA), polycaprolactone (PCL)), polyanhydrides, polyphosphazenes and polyurethanes. These synthetic polymers are economically cheaper, biodegradable, stable with a longer shelf life and scalable. However, the possibility of rejection due to immune responses is a major drawback for these synthetic polymeric scaffolds in physiological environments [39,40].

Ceramics (hydroxyapatite (HA), tricalcium phosphate (TCP), bioactive glasses and glass ceramics) possess excellent mechanical strength [41]. However, they are brittle, non-biodegradable and limited in their use for tissue regeneration [42]. Mostly, biomaterials are fabricated after complexing two or more of the above-stated materials in order to introduce desirable physicochemical and biological properties [43,44].

**Table 1:** Current state of materials and fabrication technologies for tissue engineering.

Types of Biomaterials	Fabrication Method	Properties	Applications	Reference
Chitosan/HA/bioglass Scaffolds	Freeze drying	Improved biomechanical properties and in-vitro biodegradability	Bone regenerations, implants	[45]
Silk fibroin (SF) and chitosan (CS) scaffolds	Freeze drying	The effect of different ratio in blend was optimized	Improved cartilage regeneration	[46]
SF/gelatin G, chondritin sulfate C, hyaluronic acid H scaffolds.	Freeze drying	High porosity, Enhanced proliferation and chondrogenic differentiation	Cartilage tissue engineering, bone marrow mesenchymal stem cells, BMSC	[47]
SF-polydopamine-E7 peptide functionalized scaffolds	Electrospun	Improved hydrophilicity, cellular proliferation and differentiation	BMSC, bone tissue engineering	[48]
SF/Graphene oxide functionalized by BMP-2 peptide	Electrospun	Coated scaffold improved biological properties and bone regeneration	BMSCs, sized bone defects	[49]
Fe_3_O_4/_Mesoporous bioactive glass/PCL	3-D bioprinting	Sustained drug delivery with excellent magnetic heating ability, proliferation and mineralization of ECM	Local anticancer drug delivery	[50]
Tricalcium phosphate	3-D printing	Ability to replicate cortico-cancellous alveolar bone architecture with dual layers including compact and porous structures	Bone tissue engineering	[51]
PLGA and Solid lipids (Softisan 154 and Witepsol H42) as porogen materials	Solid lipid templating	Easy control of architectural properties and scalable automated production, quick porogen extraction, avoids aqueous media and use of sophisticated equipments	Cartilage tissue engineering	[52]
Artificial ECM coated PLGA scaffolds	Solid lipid templating	Enabled suitable growth, proliferation and ECM metabolism of dermal fibroblasts for 14-days	3-D substrate for Human dermal fibroblasts	[53]

## 4. Lipids Used in Drug Delivery Systems

Lipid-based drug delivery provides a suitable means of releasing drugs in a site-specific and controlled manner. Lipids have a higher degree of biocompatibility and are available in different molecular weights. These are particularly suitable for poorly water-soluble drugs [54]. Lipids consist of both hydrophilic and hydrophobic portions and can self-assemble to form a variety of structures [55]. Cationic lipids are suitable for the delivery of drugs and genes [56]. Cationic lipids used for gene delivery consist of a positively charged head group and a linker that joins the head with a hydrophobic chain [57]. Cationic lipids have particularly gained importance for the delivery of nucleic acids. Nucleic acids cannot be directly taken up by the cells and need facilitated transport. Secondly, nucleic acids are negatively charged and have very poor affinity for cell membranes. Condensation with cationic lipids was a suitable means of delivery that provided protection and increased their half-life [58]. Cationic lipids are preferred because they interact with the negatively charged plasma membrane that leads to enhanced cellular uptake [59]. However, cationic lipids may interact with serum protein and form aggregates [60]. Cationic liposomes are also rapidly cleared by the reticuloendothelial system (RES) [61]. Liposomes made up of anionic or neutral lipids survive up to 5 times longer [62] in blood circulation compared to cationic lipids [63]. Liposomes made up of anionic lipids have better stability and promote a pH-responsive release of drug in an acidic environment. These liposomes have good stability at neutral pH and release the drug at acidic pH [64].

Lipid-based excipients such as fatty acids, non-ionic surfactants and glycerides also act as permeability enhancers by increasing the fluidity of the plasma membrane. Some medium-chain glycerides also inhibit the efflux pump [65]. Different types of lipids have reactive carboxylic acid groups such as fatty acids that can be directly conjugated with drugs through amide or ester linkages. Some cholesterol-based steroids have also been conjugated with drugs to enable enhanced penetration across the plasma membrane with cellular uptake [66].

Triglycerides are also effective for drug delivery by conjugation with drug molecules. Triglycerides are formed by a combination of glycerol and three fatty acids linked together via ester linkage. Replaced fatty acids at position two with the drug utilize the advantage of a triglyceride pathway. In this way, drug hydrolyzed in the gastrointestinal (GI) lumen form free fatty acids and result in enhanced absorption [67,68]. Phospholipids are also used in drug delivery by conjugation with the drug through a phosphate group linkage [69] or attachment of a glycerol backbone [70]. These lipid–drug conjugates significantly increased the bioavailability of orally administered drugs by avoiding premature hydrolysis and increased membrane permeability [71].

## 5. Lipid-Based Delivery Systems in Regenerative Medicine

Trauma occurs readily in the human body. Skin, being the largest organ of the human body, is constantly exposed, making it highly vulnerable to injury. In addition, the incidence of chronic trauma caused by obesity, diabetes, and arteriovenous blood supply deficiency has increased significantly with age [72].

Most substances in use as regenerative agents are polar, hydrophilic macromolecules. Thus, they do not easily penetrate natural biological barriers (e.g., the skin, vascular endothelium, and blood–brain barrier) to enter the injured site, thereby limiting the therapeutic effect.

Nanoscale lipid preparations include liposomes, lipid nanoparticles, nano-emulsions, and micelles. Lipid nano-formulations can encapsulate hydrophilic and lipophilic regenerative agents that improve their bioavailability. Targeting can be achieved through surface modification so that the drugs accumulate in a specific area, resulting in reduced toxicity and increased efficiency. In recent years, many studies that applied lipid nano-formulations to repair tissue damage have achieved good results.

Kazemi et al. [73] used the spontaneous emulsification method to prepare a nano-emulsion for wound treatment with lavender essential oil and licorice extract. Treatment with the nano-emulsion increased the expression of transforming growth factor-β (TGF-β), increased the expressions of type I and type III collagen genes, accelerated the formation of granular tissue and collagen, reduced the degree of lipid peroxidation, and increased the activities of superoxide dismutase (SOD) and glutathione peroxidase (GPx). These results demonstrate that the nano-emulsion prepared from lavender essential oil and licorice extract shows promise for wound repair.

Fan et al. [74] prepared a micelle preparation containing curcumin and hyaluronic acid. The prepared micelles reduced the expressions of related cytokines and vascular endothelial growth factor and significantly reduced the degree of edema in arthritic rats. Reducing the friction between the cartilage surfaces around the joints can protect the cartilage from damage caused by rheumatoid arthritis. Todorovic et al. [75] evaluated the effect of CoQ_10_ encapsulation in nanoliposomes on wound healing after tooth extraction through tissue biochemical (myeloperoxidase activity and nitric oxide concentration) and histopathological analyses. The results showed that the encapsulation of CoQ_10_ in nanoliposomes significantly reduced wound inflammation after tooth extraction in rats and enhanced the ability of CoQ_10_ to promote wound healing. Wang et al. [76] loaded liposomes with blue copper peptide (GHK-Cu) and analyzed their effects on the proliferation of mouse umbilical vein endothelial cells and scald wound healing. The results showed that the GHK-Cu liposomes enhanced the expressions of vascular endothelial growth factor (VEGF), fibroblast growth factor-2 (FGF-2), and the cell-cycle-related proteins CDK4 and CyclinD1. In the scald mouse model, treatment with GHK-Cu liposomes had a better effect on angiogenesis in burned skin compared to treatment with free GHK-Cu; GHK-Cu liposome treatment enhanced the CD31 and Ki67 signals in mice and shortened the wound healing time after injury to 14 days. Many studies have investigated the ability of lipid nano-formulations to repair tissue damage [77,78,79,80]. This progress has led to lipid nano-formulations that play an increasingly important role in tissue repair.

### 5.1. Liposomes

Liposomes, which are double-layered vesicles constructed from amphiphilic molecules including phospholipids, have become important drug carriers [81]. Liposomes are non-toxic, biodegradable, biocompatible, and able to simultaneously encapsulate hydrophilic and lipophilic drugs. By encapsulating the drugs, liposomes protect the drugs and allow for sustained drug release. Hydrophilic drugs can be encapsulated in the inner cavity of the liposome, while hydrophobic drugs can be loaded in the phospholipid double layer [82,83]. The advantages and disadvantages of liposomes as drug delivery carriers are summarized in Table 2. Moreover, the application of liposomes as carriers for drugs in regenerative medicine is reviewed based on four aspects: the treatment of spinal cord injury, neuron damage, thrombosis, and skin wounds.

#### 5.1.1. Application of Liposomes in the Treatment of Spinal Cord Injury

Clinically, spinal cord injury is a highly disabling disease. With the development of modern industry, the number of people suffering from spinal cord injury has increased [90]. After spinal cord injury, patients often have various secondary diseases, such as hemorrhage, edema, neuronal apoptosis, and necrosis, which further damage nervous system function [91]. Physically and psychologically, patients and their families both bear the substantial burden of spinal cord injury [92,93]. The research and development of drugs for repairing spinal cord injury is ongoing, and liposomes have been widely applied as delivery systems for these drugs. Wang et al. [94] constructed tetrapeptide (CAQK)-modified liposomes, used the liposomes to encapsulate docetaxel (DTX) and brain-derived neurotrophic factor (BDNF), and mixed the loaded liposomes with heparin (HP) and an acidic fibroblast growth factor (aFGF) hydrogel to obtain CAQK-LIP-GFs/DTX@HP. CAQK-LIP-GFs/DTX@HP delivered the drugs to the injury site. The combination of GFs and DTX supported nerve regeneration by improving the survival and plasticity of neurons, providing a promising therapeutic strategy for the clinical treatment of spinal cord injury.

Gao et al. [95] prepared PEG-TAE-modified PLGA polymer liposomes and loaded them with cyclosporin A, which allowed cyclosporin A to penetrate the blood–spinal cord barrier and effectively treat spinal cord injury. In animal experiments, the cyclosporin A-loaded liposomes resulted in a higher concentration of cyclosporin A in the spinal cord compared to the free drug after injection through the tail vein, and loading cyclosporin A into liposomes significantly increased the expression of the growth-related protein GAP43 and greatly increased the number of GAP43-stained neurons.

#### 5.1.2. Application of Liposomes in the Treatment of Neuron Damage

Alzheimer’s disease (AD) is a chronic neurodegenerative disease. The typical manifestation of AD is the accumulation of β-amyloid (Aβ), which produces senile plaques in the cerebral cortex and hippocampus and leads to synaptic dysfunction and neurofibril formation. Hyperphosphorylated tau that generates neurofibrillary tangles (NFTs) leads to synaptic dysfunction [96,97]. The degenerative neuropathy of AD poses a substantial challenge to the medical community. Ongoing research aims to relieve the symptoms of AD through neuron repair.

Yue et al. [98] encapsulated the glial cell-derived neurotrophic factor (GDNF) plasmid gene in PEG-modified liposomes to create PLs-GDNF-MBs. Treatment with PLs-GDNF-MBs alleviated behavioral defects in Parkinson’s rats and increased the expressions of GDNF and Nurr1 to protect neurons. Cheng et al. [99] developed epigallocatechin-3-gallate (EGCG)-loaded liposomes and demonstrated that the EGCG-loaded liposomes could activate microglia in a rat model of Parkinson’s syndrome in vivo and exert a neuroprotective effect. Kuo et al. [100] prepared serotonin (SM)- and apolipoprotein E (APOE)-modified liposomes and loaded them with nerve growth factor (NGF). The resulting NGF-SM-APOE-LIP liposomes facilitated the passage of NGF through the blood–brain barrier. Compared to free NGF, NGF-SM-APOE-LIP upregulated the expression of phosphorylated neurotrophic tyrosine kinase receptor 1 in cholinergic neurons and significantly improved their survival. Treatment with NGF-SM-APOE-LIP also reduced the secretion of acetylcholinesterase and malondialdehyde in the brains of rats, thereby hindering apoptosis in hippocampal neurons.

#### 5.1.3. Application of Liposomes in the Treatment of Thrombosis

Thrombosis is an obstruction of blood flow due to the destruction of the hemostatic regulation mechanism, leading to myocardial infarction, stroke, or pulmonary embolism [101]. Thrombolytic therapy uses thrombolytic drugs such as streptokinase, urokinase, and tissue-type plasminogen activator to dissolve the thrombus and restore the blood supply to tissues. Streptokinase, extracted from Streptococcus, is immunogenic and has a short half-life, while urokinase, which is not immunogenic, has a very short half-life and no ability to target the thrombus [102]. Tissue-type plasminogen activator is a thrombolytic drug with high thrombus affinity; however, its adverse effects and high cost limit its application [103]. Therefore, it could be of great significance to develop drug delivery systems that can deliver thrombolytic drugs directly to the thrombus site. Liposomes are suitable candidates for this purpose because of their targeting ability.

Vaidya et al. [104] incorporated streptokinase into highly selective target-sensitive liposomes. In vitro studies demonstrated that the liposomes could release streptokinase after binding to activated platelets. An intravital microscopy study of a thrombus mouse model showed that the liposomes preferentially accumulated in the thrombus area. In vivo studies showed that the target-sensitive liposomes produced a 28.27% ± 1.56% reduction in the thrombus, greater than the reduction achieved by streptokinase solution (17.18% ± 1.23%). Moreover, liposome treatment reduced the clot dissolution time compared to treatment with streptokinase solution.

Zhang et al. [105] loaded urokinase into liposomes modified with PEG and cRGD polypeptide. Flow cytometry analysis showed that the cRGD-modified liposomes bound to activated platelets but not resting platelets. In vitro release studies demonstrated that approximately 60% of urokinase was stably and continuously released within five hours, and a constant, high local drug concentration was maintained. In vivo thrombolysis studies using a mouse mesenteric thrombosis model indicated that the thrombolytic effect of the cRGD-modified liposomes was nearly four times that of free urokinase.

#### 5.1.4. Application of Liposomes in the Treatment of Skin Wounds

The skin is the largest organ of the human body, acting as an important barrier and performing critical functions related to immunity, sensation, and protection. Due to its exposure to the external environment, the skin is susceptible to various types of skin injuries. Liposomes have the following significant advantages in wound repair: (1) liposomes are non-toxic and have good compatibility with skin, creating a moist environment that is favorable for wound repair [106]; (2) liposomes can effectively encapsulate drugs and inhibit their degradation [107]; and (3) liposomes possess sustained-release characteristics, reducing the frequency of administration [108]. Based on these advantages, liposomes have been widely used in wound treatment and skin regeneration.

##### Skin Repair for Burns

Thermal burns, which mainly include fire burns and scalds, are one of the most common types of trauma affecting humans [109,110]. Burn wounds are extremely susceptible to bacterial infections. The infection of untreated burn wounds causes delayed healing and scar formation and may lead to bacteremia, sepsis, or multiple organ dysfunction syndrome, which can seriously affect the physical and psychological health of the patient [111]. Therefore, strategies that improve the healing rate of burns and reducing scarring are important.

Purslane glycoside (MA) is a saponin extracted from Centella asiatica. MA has good antibacterial, anti-inflammatory, and antioxidant effects, and it can also promote cell growth and proliferation [112,113]. However, the high hydrophilicity and low permeability of MA through skin tissue limit its topical application. Liposomes can facilitate the permeation of drugs into the epidermis. Liu et al. [114] designed and prepared PEG-modified MA liposomes via a two-step emulsification method and assayed them for in vitro skin permeation, skin distribution, and burn wound healing tests. The transdermal performance and wound healing effect of the MA double-emulsion liposomes were better than those of the MA solution. The common liposome does not maintain sufficient adhesion to the wound surface. Polyethylene glycol-polycaprolactone-polyethylene glycol (PEG-PCL-PEG; PECE), a biodegradable temperature-responsive copolymer, was synthesized and used to improve the adhesion properties of MA liposomes [115]. In secondary burn experiment in rats, the PECE-modified MA liposomes had a better wound contraction effect than MA liposomes.

Using silk fibroin as the hydrogel core, Xu et al. [116] prepared a new liposome that can efficiently encapsulate bFGF. The carrier significantly improved the stability of bFGF in the wound fluid, and the cell proliferation activity and wound healing activity were maintained over 72 h. In addition, the liposomes with hydrogel cores effectively induced angiogenesis. In another study, azone was added as a penetration enhancer to liposomes encapsulating bFGF to construct skin-permeable liposomes (SP-bFGF-SF-LIP). SP-bFGF-SF-LIP significantly increased the concentration of bFGF in the dermis. Moreover, treatment with SP-bFGF-SF-LIP significantly improved the morphology of wound hair follicles, and the burn wound was repaired with hair regrowth [117].

##### Skin Repair for Ultraviolet B (UVB) Radiation Damage

UVB radiation is a common cause of skin damage. The skin that is frequently exposed to UVB radiation produces active oxygen, which destroys the skin’s normal antioxidant defense system and often induces inflammation [118,119,120,121]. UVB radiation can also directly cause DNA damage, as indicated by the generation of cyclobutane pyrimidine dimer (CPD) and 8-hydroxy-2′-deoxyguanosine (8OHdG), which are often used as markers to investigate the degree of DNA damage [122,123,124,125]. Although traditional physical and chemical sunscreens are widely used, new methods are still needed to cope with UVB radiation-induced damage.

Trehalose is a natural non-reducing disaccharide that cannot easily penetrate the skin. Emanuele [55] encapsulated trehalose in liposomes and found that, compared to commonly used photoprotective compounds such as L-carnosine, L-(+)-lysergic acid, and L-ascorbic acid, trehalose-loaded liposomes significantly reduced the levels of CPD and 8OHdG along with protein carbonylation in cells, indicating that the liposomes show promise for treating skin damage caused by UVB radiation.

Zhang et al. [126] prepared glycyrrhizin liposomes (GLs) with low cytotoxicity and a significant inhibitory effect on melanin. By regulating the expression of inflammatory factors (TNF-α, IL-6, and IL-10), the GLs alleviated the skin damage caused by UVB radiation.

Spanidi et al. [127] prepared cyclodextrin liposomes (CRPP) containing propolis polyphenols. CRPP can protect human permanent keratinocytes (HaCaT) from the mutagenic effects of ultraviolet radiation and reduce the content of ultraviolet radiation-induced protein carbonyls. The application of CRPP to recombinant skin tissues also demonstrated that CRPP can reduce the expression of matrix metalloproteinases in mRNA along with protein levels, indicating that CRPP can resist UVB damage.

##### Skin Repair for Diabetic Ulcers

Unhealed chronic wounds plague many patients and health systems in various countries [128]. Due to normal aging along with increases in diabetes and obesity, the incidence of chronic trauma is increasing.

Diabetic ulcers are difficult to treat effectively due to the complexity of the wound’s conditions [129,130]. According to reports, 15% of diabetic patients will suffer from diabetic foot ulcers, and 85% of diabetic foot ulcers will be amputated [131]. Thus, effective treatments for diabetic ulcers are urgently needed, and liposomes have potential applications in this area.

The main symptoms of diabetic ulcers are decreased peripheral blood perfusion and slow wound healing [132]; thus, increasing blood microcirculation and improving oxygen transport are beneficial for ulcer wounds. Fukui et al. [133] loaded hemoglobin, which has high affinity for O_2_, into liposomes. In a mouse model of diabetes injury (dB/dB), liposome-encapsulated hemoglobin significantly increased surface blood flow and inhibited inflammatory factors. The resulting healing speed of skin wounds was comparable to that of normal mice.

Choi et al. [134] coupled low-molecular-weight protamine to the n-termini of EGF, PDGF-A, and IGF-1, combined these substances with hyaluronic acid, and then encapsulated them into cationic elastic liposomes. The resulting GF-containing liposomes significantly improved the wound healing rate in a diabetic mouse model. The maximum rates of wound shrinkage were decreased by 65% and 58%, compared with those obtained with drug-loaded cationic and natural growth factor complexes, respectively.

In another study, miR-132 was found to have an inhibitory effect on inflammation; the expression of miR-132 was upregulated during normal skin wound healing, while the expression of miR-132 is decreased in the epidermis of diabetic ulcer wounds [135]. The local injection of liposomes containing an encapsulated miR-132 mimic significantly increased the expression of miR-132 and accelerated wound healing.

The repair of damage has always been a clinically difficult problem, and current treatment methods cannot always achieve good therapeutic effects. In recent years, in-depth studies on liposomal nano-formulations have generated new treatment methods for damage repair. Liposome carriers protect the encapsulated drug from degradation while also improving the drug’s ability to cross biological barriers. The good biocompatibility and targeted drug delivery ability facilitate the entrance of the encapsulated drugs into the target tissues. However, liposomal nano-formulations have certain limitations. While their effectiveness has been confirmed in numerous basic research studies, liposomal nano-formulations remain difficult to get into industrial production. As production techniques advance, liposomes are expected to enter the market in this field of regenerative medicine.

### 5.2. Lipid Nanoparticles

Two types of lipid-based nanoparticles with a solid matrix have been reported. The physiological lipids that are solid at room, human body and skin temperatures make up the majority of solid lipid nanoparticles (SLNs), also called first-generation lipid nanoparticles. Various lipophilic and hydrophilic natural and synthetic therapeutic moieties can be encapsulated within the lipid melt by utilizing suitable fabrication methods such as high-pressure homogenization including hot homogenization, the cold homogenization technique, the high shear homogenization and ultrasonication techniques, microemulsion, the membrane contactor technique, the phase inversion temperature technique, the coacervation technique, double emulsion, and the emulsification solvent evaporation and solvent diffusion techniques [136,137]. LNs have been proposed as an alternative carrier to numerous other colloidal drug delivery systems for many reasons, including submicron size range (50–1000 nm) [138], avoidance of organic solvents utilization, use of lipids that are GRAS (generally regarded as safe) and cheap fabrication processes. The second-generation lipid nanoparticles, also called nanostructured lipid carriers (NLCs), were reported to overcome some limitations of SLNs, including less loading capacity due to a highly ordered crystalline structure and drug expulsion on storage. These nanocarriers are made up of a less ordered lipid matrix that can accommodate more drug compared to SLNs.

Various research findings of LBNs have been reported with superior performance of loaded drugs for injured tissue repair, burns, wound healing, and better antibacterial and antifungal properties [139]. Wound healing is a complex biochemical process immediately required for the restoration of the structure and function of any damaged tissue. Any skin damage activates series of processes including blood clotting, inflammation, tissue repair and remodeling. The chronic and acute wounds present a major challenge for research based on the stages and complexities of wounds [140].

The topical applications of lipid-based nanoparticles have been explored in depth due to the occlusive nature of formulations. In vivo and ex vivo biodistribution studies of NLCs radiolabeled with 99 mTc were performed by Vairo et al. Their developed NLCs were safe, remained on the wounds and were not absorbed for at least 24 h [141].

Curcumin solid lipid nanoparticles were loaded in sponges to target the buccal mucosa, were developed as a solid dosage form to maintain their integrity and sustained release for 14–15 h [84].

Sandri et al. developed wound dressings with SLNs based on chondroitin sulfate and sodium hyaluronate and loaded with silver sulfadiazine associated with platelet lysate. The prepared dressings exhibited good mechanical, hydration and bioadhesion properties suitable for the treatment of skin lesions [142,143].

Saporito et al. developed NLCs (range 220–300 nm), with cocoa butter as the solid lipid and olive oil as the liquid lipid, encapsulated with essential oils. The lipid constructs exerted a synergistic effect that promoted antimicrobial properties and wound healing capabilities when loaded with eucalyptus oil or rosemary oil when tested in a rat burn model [144].

### 5.3. Lipid-Core Micelles

Micelles are aggregates of globular amphiphilic molecules or amphiphilic block copolymers that are thermodynamically stable. They are usually in the diameter range of 10–100 nm. Polymeric micelles and reverse micelles are particularly relevant in drug delivery systems. Some of the advantages of micelles include prolonged release, encapsulation of hydrophilic or hydrophobic drugs, increased solubility and bioavailability, lowering the dosage and the frequency of administration, and lower toxicities in comparison with conventional drugs. Despite all of these above-mentioned benefits, micelles are seldom used in regenerative medicine [145].

#### 5.3.1. Micelles in Anti-Angiogenic Activity

Angiogenesis, the formation of new blood vessels, is a crucial step in the progression of cancerous diseases. The tumor cells need blood vessels for tumor growth, invasion and metastasis. Studies have shown that anti-angiogenesis can be used as a therapy for cancer treatment. Most anti-angiogenic therapies come with toxicities such as bleeding, thrombosis, lymphopenia and immunomodulation. However, the use of targeted drug delivery systems can be used to mitigate the toxicities caused by the treatments [146]. Most of the angiogenic therapeutic agents undergo degradation at physiological conditions. Encapsulation in a colloidal system such as micelles can protect the drugs from degradation.

Mandraccia and Tripodo et al. used polymeric micelles to deliver curcumin and celecoxib, both of which are highly hydrophobic drugs. They synthesized a polymer containing inulin (IN) and vitamin E (VITE) for hydrophilic and hydrophobic moieties, respectively, which can self-assemble as micelles and encapsulate highly hydrophobic drugs such as curcumin and celecoxib. Some of the advantages of this delivery system include the lack of charge on the surface and their very small size, which allow them to cross the cell membrane more easily. Their studies showed a sustained release of curcumin and celecoxib for 7 days under sink conditions. Moreover, the INVITE polymeric micelles loaded with curcumin and celecoxib had significant anti-angiogenesis and tumor shrinkage. Their INVITE micelles were efficient at increasing the water solubility of both the drugs and, since both inulin and vitamin E are of natural origin, the micelles are highly biocompatible [147].

#### 5.3.2. Micelles in Bone Regeneration

Lipid-based micelles can be used to deliver synthetic glucocorticoids to aid in the differentiation of bone-marrow-derived mesenchymal stem cells (BMSCs). BMSCs have shown potential in bone differentiation both in animal and human models [85,86,87,88,89,148,149,150,151,152]. Dexamethasone (Dex) is a poorly soluble glucocorticoid that can induce bone regeneration by triggering chemokine and calcium signaling. Dexamethasone has shown stimulatory effects both in early and late phases of bone differentiation, ultimately directing the cells to mineralization of nodules. The modulation of nuclear receptors was observed with dexamethasone, which leads to transduction mechanisms ultimately leading to bone growth. Dex also regulates Runx2 by the activation of the FHL2/β-catenin-mediated transcription pathway via the activation of both TAZ and MPK-1 [90,91,92,153,154,155]. It also showed an increase in the cells containing alkaline phosphatase, which is a marker of osteogenic activity. Factors such as donor species, degree of cell differentiation, dosage, dose duration and dosing regimen will play a role in dexamethasone regulation [156].

Santo et al. synthesized dexamethasone-loaded gelatin micelles that showed a pH-dependent release profile for dexamethasone. They also observed that these micelles were internalized into different cell types with an efficiency of between 65 and 100%. To test the route of internalization, the researchers have blocked the endocytic pathway and observed a significant decrease in micelle uptake, indicating that the particles are internalized via endocytosis. A dose-dependent response of the cells to dexamethasone was observed during in vitro studies. Lower doses of dexamethasone showed a cell-killing effect; after the complete release of dex, the cells regained their proliferative activity. They have also noticed increased alkaline phosphatase levels in the cells and depositions of the mineralized matrix. The extent of bone regeneration was dependent upon the dosage of dex-micelles incubated with the stem cells and the amount of stem cells that were seeded onto gelatin scaffolds. The new tissue formed had high levels of bone extracellular matrix organization, unlike treatment with empty micelles or free dexamethasone, which showed fibrous tissue deposition. Santo et al. were able to regenerate bone by altering properties such as the dosage of delivery systems to achieve high levels of tissue regeneration in a cost-effective manner [156].

**Figure 3:**
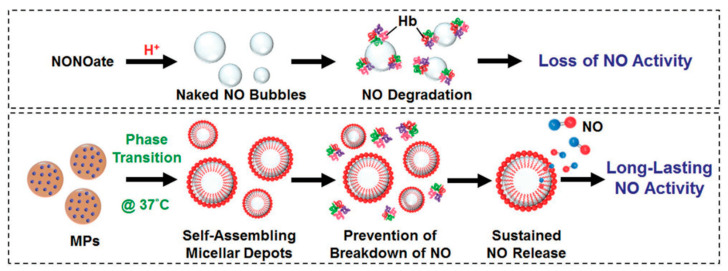
In situ generation of NO from the micellar depots after phase transition in comparison to naked NO formed the NONOate donor [157].

Lin et al. have delivered nitric oxide using a lipid-based microparticle system and increased its half-life of NO using a micelle-based delivery system. In theory, prolonged exposure to elevated NO levels in the body causes enhanced osteoblast activity resulting in osteogenesis. Diethylenetriamine diazeniumdiolate (NONOate), a NO donor, was encapsulated in the micelles, which released NO in a sustained manner (Figure 3). The NO half-life was extended from 20.6 s in NONOate donor to 21.6 min from the micelles. In vivo studies in the osteoporosis rat model have shown decreased bone turnover and, therefore, thicker trabecular bones along with denser networks and lower marrow fat levels [157]. As illustrated in Figure 3, the microparticles injected subcutaneously undergo a phase transition and form micellar depots that release NO from the donor.

#### 5.3.3. Micelles in Myocardial Infarction

Nguyen et al. have used micelles to target matrix metalloproteinases (MMPs), which are upregulated in myocardium, undergoing a myocardial infarction (MI). MMP-targeting peptide (MMP-TP)-loaded micelles have been used to target MMPs in the myocardium. In vitro studies on U-937 cells have shown MMP-TP micelles to be better at binding to activated cells than the plain micelles. Early research has shown that MMPs have an important role in remodeling and restructuring the extra cellular matrix after MI [158,159]. This is an inflammatory response that Nguyen et al. chose to exploit. MMPs such as MMP-2 and MMP-9 are key players in remodeling the left ventricle associated with the degradation of collagen, laminin, fibronectin and elastin [160]. The micelles were lipid based and made of phosphotidyl ethanolamines such as DSPE. In vivo studies involving C57BL/6 mice showed increased accumulation of MMP-TP micelles in the area of ischemia, resulting in cardiac regeneration using MMP-TP micelles. These micelles were efficient at the delivery of reprogramming factors that can genetically modify macrophages and help in tissue remodeling [161].

Wang et al. have reported on shortening the prolonged inflammation after MI using CCR2 inhibitor-loaded micelles. Monocytes are recruited at the infarct via the CCR2 chemokine receptor along a CCL2 gradient. The micelles were loaded with a small-molecule CCR2 antagonist, and the surface of the micelles was functionalized with anti-CCR2 antibody. They observed an eight-fold increase in the binding of the micelles with the CCR2 expressing RAW 264.7 monocytes compared to plain micelles. In vivo studies in a mouse model showed the loaded and functionalized micelles to reduce the Ly6C inflammatory cells to 3% and further decrease the infarct size compared to PBS and non-targeted micelles [162]. Li et al. delivered lipid-based puerarin, which has a protective property against MI due to its regulation of mitochondria, whereas free puerarin did not targeted to ischemic cardiomyocytes [163].

### 5.4. Colloids

Colloidal gels are suitable for use in regenerative medicines due to their higher water contents, self-assembly and flexible mechanical properties. Colloidal gels having a paste-like appearance have shown promise in craniofacial bone regeneration. In a study of hydroxyapatite-based colloidal nanoparticles containing decellularized cartilage (DCC), the demineralized bone matrix (DMB) was prepared with hyaluronic acid to form a colloidal gel having suitable rheological properties. The results showed an 82% higher bone regeneration rate. DCC could be a potential material for bone regeneration application, and colloidal gel may be a promising delivery tool for craniofacial application [164].

Regenerative medicines are effective for the treatment of bone loss, particularly for diseases such as osteoporosis. In another study, colloidal gel was prepared for osteoporotic bone regeneration made up of bioactive glass particles and bisphosphonate functionalized gelatin. This colloidal gel of gelatin nanoparticles showed greater cell proliferation ability in vitro and regeneration of osteoporotic bone in vivo [165]. Injectable regenerative medicines are a suitable alternative to invasive surgeries. Colloidal gels were prepared from anionic hydroxyapatite (HaP) nanoparticles and cationic PLGA nanoparticles. The colloidal gels are stabilized by electrostatic forces that can be disrupted to facilitate extrusion. The colloidal gel showed a 3D porous structure on SEM analysis. This colloidal gel of Hap/PLGA nanoparticles provided a suitable means of intravenously delivering regenerative bone materials [166].

Colloidal hydrogels were prepared from mesoporous silica nanoparticles embedded with PEG-PLGA- Poly N-(isopropylacrylamide) hydrogel for the co-delivery of microRNA-222 and aspirin. Aspirin is known to stimulate bone formation, and miR222 is known to induce bone mesenchymal stem cell differentiation. In a rat model with a mandibular bone defect, co-delivery of aspirin and miR222 in a colloidal gel resulted in enhanced bone formation and neurogenesis. The results indicate that a colloidal gel of aspirin and miR222 has potential for bone tissue engineering [167].

Colloidal hydrogels are formed by the aggregation of micro and nanoparticles through physical or electrostatic interactions [168]. Carboxyl modification of synthetic polymers mimics the non-collagenous proteins. This biomimetic approach can be utilized to prepare the carboxyl modification of PLGA nanocomposites. These ionic colloids improved bone repair by enhancing mechanical properties and result in osteogenic differentiation. This led to enhanced bone density with less fibrous tissues in rabbit models. These ionic colloids may serve as suitable biomimetic scaffolding to rebuild bone tissues [169].

Simvastatin is an antihyperlipidemic drug but also has osteoanabolic effects and can be used in bone regeneration. However, for bone generation, it must be delivered to the targeted site to avoid undesirable side effects. Lipid nanoparticles were prepared using emulsifying lipids and glyceryl monooleate. Colloidal lipid nanoparticles were loaded with simvastatin. Studies for bone regeneration were carried out on osteoblastic (bone forming) and osteoclastic (reabsorb bone) cells. The results showed that simvastatin nanoparticles support osteoblastic cell differentiation and inhibit osteoclastic cells. The overall work demonstrated that these lipid nanoparticles loaded with simvastatin could be used for bone regeneration applications [170]. Polymers such as PLGA have biodegradable and biocompatible properties. PLGA nanoparticles were loaded with bone morphogenetic protein (BMP-2), and colloidal solution was prepared by a double emulsion, solvent evaporation technique. The colloidal nanoparticles with sizes of 100–500 nm were prepared and analyzed for colloidal properties. The effects of BMP-2-loaded nanoparticles were studied for the osteogenic differentiation of alveolar bone and showed enhanced efficacy in vitro [171]. Colloidal hydrogels are also a suitable vehicle for tissue engineering applications. Colloidal gels were prepared by a reaction of a carboxyl-modified aniline dimer and gelatin. Dexamethasone was loaded as a model drug. The rate of hydrogel degradation was decreased by increasing aniline dimer concentration. The electroactivity of colloidal hydrogel was determined by using cyclic voltammetry. The results showed that stearic hinderance prevented the hydrogel from achieving a fully oxidized state. This colloidal gel may be suitable for neural tissue engineering applications [172].

Overall, colloids and colloidal hydrogels are suitable vehicles for bone regeneration and tissue engineering applications due to tunable properties and provide a platform for regenerative medicines.

## 6. Concluding Remarks

Nowadays, regenerative medicine is based mostly on tissue engineering and stem cell therapies. Although stem cells can be obtained, it is impossible to use them for therapeutic purposes by transplanting the cells prepared. However, based on knowledge from a variety of sources, cells and other cell-based therapies are not fully effective, unless an environment that promotes their proliferation and differentiation can be created. Thus, there remains a need for the development of strategies that can provide environmental control, such as delivery systems. The development of these strategies should significantly contribute to the development of regenerative technologies. The use of stem cells and soluble factors delivered by specific carriers and scaffolds may have expanded clinical applications in the future. However, more work needs to be carried out, using both in vitro and in in vivo models, before these therapies can be considered for clinical trials. Specifically, collaboration between pharmaceutical, biological, material, and clinical scientists will be required for further progress in the field. The lipid-based nanocarriers for regenerative medicine can open a lot of possibilities to overcome the limitations of cell-based therapies. They can provide more affordable and scalable technologies in the realm of regenerative medicine. Moreover, the nanocarrier systems discussed in this paper can be used to deliver appropriate growth factors to the injury site and create an environment conducive to the regeneration of the cells. Certainly, there is potential for regenerative medicine to change in the field of health care from a reactive endeavor to a preventative and restorative endeavor. After evaluating the pros and cons of both lipid-based delivery systems and cell-based therapies, the authors suggest that a combination approach can better aid regeneration at the injury site.

This review has provided some insights into the applications of lipid-based delivery systems that enhance tissue regeneration and inspire continued work in this field.

## Figures and Tables

**Table 2:** Advantages and drawbacks of liposomes as carriers in regenerative medicine.

Advantages	Drawbacks
Biocompatibility [84]	High production cost [85,86]
Amphiphilic drug loading [83]	
Sustained drug release effect [87]	Difficulty in transportation and storage [85]
Targeting effect [88]	
Low toxicity [89]	
